# Co-occurrence pattern of congeneric tree species provides conflicting evidence for competition relatedness hypothesis

**DOI:** 10.7717/peerj.12150

**Published:** 2021-11-02

**Authors:** Shuntaro Watanabe, Yuri Maesako

**Affiliations:** 1 Graduate School of Science and Engineering, Kagoshima University, Kagoshima, Japan; 2 Graduate School of Human Environment, Osaka Sangyo University, Osaka, Japan

**Keywords:** Competition-relatedness hypothesis, Species-to-genus ratio, Reproductive interference, Warm-temperate evergreen forest, Kasugayama forest reserve

## Abstract

In plants, negative reproductive interaction among closely related species (*i.e.*, reproductive interference) is known to hamper the coexistence of congeneric species while facilitation can increase species persistence. Since reproductive interference in plants may occur through interspecific pollination, the effective range of reproductive interference may reflects the spatial range of interspecific pollination. Therefore, we hypothesized that the coexistence of congeners on a small spatial scale would be less likely to occur by chance but that such coexistence would be likely to occur on a scale larger than interspecific pollination frequently occur. In the present study, we tested this hypothesis using spatially explicit woody plant survey data. Contrary to our prediction, congeneric tree species often coexisted at the finest spatial scale and significant exclusive distribution was not detected. Our results suggest that cooccurrence of congeneric tree species is not structured by reproductive interference, and they indicate the need for further research to explore the factors that mitigate the effects of reproductive interference.

## Introduction

Understanding how biotic interaction affects species composition and distribution is a major ongoing challenge in community ecology. Among biotic interactions, competition is the most important and well-studied interaction (Goldberg & Barton, 1992) although facilitation plays a major role in supporting biodiversity and shaping community structure (Losapio et al., 2021). A common hypothesis related to the role of competition in community assembly, termed the competition-relatedness hypothesis (CRH; Cahill et al., 2008), states that closely related species compete more intensely than distantly related species, which hypothetically limits the ability of closely related species to coexist (Webb et al., 2002; Slingsby & Verboom, 2006; Prinzing et al., 2008; reviewed by Mayfield & Levine, 2010; HilleRisLambers et al., 2012). The findings of Elton (1946), *i.e.,* that a lower number of species per genus are observed in local areas than in the entire United Kingdom, are considered as evidence for the competitive exclusion of ecologically similar congeners in local habitats. However, the CRH has also been widely discussed (Dayan & Simberloff, 2005), and empirical support for the hypothesis remains inconclusive (Webb et al., 2002; Cavender-Bares et al., 2004; reviewed by Mayfield & Levine, 2010). For plants, Cahill et al. (2008) tested the CRH using experimental data; they revealed that the relationships between phylogenetic relatedness and competitive ability differed between monocots and eudicots.

Although the evidence for the CRH in plants is mixed, recent studies have shown that negative reproductive interaction among closely related species (*i.e.,* reproductive interference) limits the coexistence of closely related species (Weber & Strauss, 2016). Reproductive interference (RI) is defined in the present study as the negative fitness effects of pollen transport among species. In plants, the effect can be generated at various stages and by various mechanisms, *e.g.*, consumption of ovules through seed set failure (Nishida et al., 2014), pollen allelopathy (Murphy, 2000), stigma clogging (Shore & Barrett, 1984) caused by interspecific pollen transfer, and production of unviable hybrids (*e.g.*, Brown & Mitchell, 2001; Takakura et al., 2009; Takakura et al., 2011; Takakura & Fujii, 2010). For this reason, the existence of reproductive interference was reported in both animal pollinated plants and wind pollinated plants (*e.g.*, Takakura et al., 2009; Takakura & Fujii, 2010). Additionally, reproductive interference involves positive frequency-dependence (Kuno, 1992). Under the positive frequency-dependence, the population growth rate of a species will decrease with decreasing relative frequency of conspecifics to heterospecifics (Takakura et al., 2009; Kishi & Nakazawa, 2013). Therefore, it can rapidly lead to the extinction of the affected species with lower population density (Kuno, 1992; Kishi & Nakazawa, 2013 ).

Previous studies of reproductive interference have provided some insight into the pattern and spatial scale of closely related species’ coexistence (Takakura et al., 2011; Whitton, Sears & Maddison, 2017; Nishida et al., 2020). Because shared recent ancestry can yield shared reproductive traits (including similarities in the timing of reproduction, mate recognition, pollination system, and gamete recognition), close relatives (*e.g.*, congeners and sister taxa) are less likely to coexist by chance on a local scale (Whitton, Sears & Maddison, 2017). Additionally, the extent of reproductive interference in plants depends on pollen transfer distance (Takakura et al., 2011). Therefore, it can be hypothesized that the coexistence of congeners on a small spatial scale is less likely to occur by chance, whereas coexistence is more likely to occur on scale larger than pollen flow distance.

In the present study, we aimed to quantitatively assess the distribution patterns of closely related woody plant species in the native forests of Japan to determine the effects of species interactions, especially reproductive interference, on forest community assembly. Using spatially explicit woody plant survey data, we tested the following predictions: (1) congeneric species do not coexist on a fine spatial scale where reproductive interference frequently occurs but coexist on a large spatial scale; and (2) on large scales where reproductive interference does not occur, congeneric species coexist while avoiding each other, resulting in an exclusive or checkerboard distribution (Connor, Collins & Simberloff, 2013).

## Materials and method

### Study site

**Figure 1:**
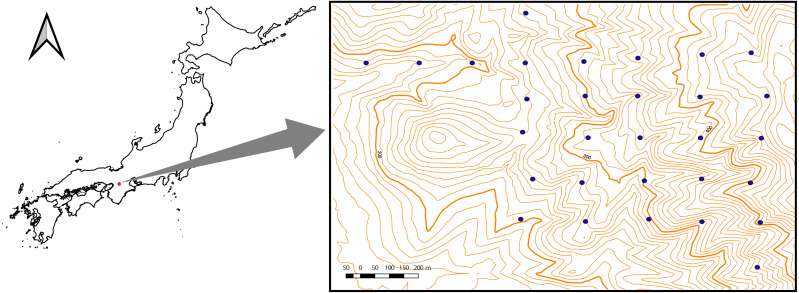
Location of the study site (1 km^2^) at Kasugayama primary forest, Nara prefecture, western Japan. The specific locations of the study plots are denoted by dots. Scale bar: 200 m.

The study area (∼1 km^2^) was located in the Kasugayama primary forest, Nara prefecture, western Japan (34′41′N135′51′E) (Fig. 1). Because the forest has been preserved as a holy site of the Kasuga Taisha shrine and logging have been prohibited there since 841 AD, the forest is considered not to have been disturbed by humans for a long time (Maesako, Nanami & Kanzaki, 2007). In the area, the mean annual temperature in 2019 was 16.3 °C and the average annual precipitation in 2019 was 1482.5 mm. The highest point of the forest is 498 m. The natural vegetation in the area is evergreen broadleaved forest (Naka, 1982); however, the deer population has recently increased in the forest, causing the spread of alien species such as *Sapium sebiferum* and *Nagia nagi* (Maesako, Nanami & Kanzaki, 2007). The field survey for this study was conducted under the permission of the Nara Park Management Office (No. 6-6).

### Field survey

Field studies were conducted from June to September 2015. In the study area, 30 transect plots (∼0.1 ha in size) at 200 m intervals were established (Fig. 1). Tree species richness was surveyed in each plot; all tree species with heights > 130 cm were recorded. Species were grouped into three life-form categories, namely trees, shrubs, or lianas, following Satake et al. (1981–1982). For categorization of small plants, species with a diameter at breast height (DBH) >10 cm on average were classified as trees while species with a DBH <10 cm were classified as shrubs.

### Interspecific competition and null model analysis

In this study, we defined congeners as closely related species based on the definition by Weber & Strauss (2016).

We employed the species-to-genus ratio (S/G ratio) as an indicator of intragenic interactions for the categorized tree, shrub, and liana species. The S/G ratio has long been used to describe community patterns and to infer levels of competitive interactions among species within genera (reviewed by Simberloff, 1970). A low S/G ratio can be interpreted as a product of strong intrageneric competition (Elton, 1946), which could limit congeneric coexistence (Darwin, 1859). First, we calculated an S/G ratio for the whole area and then tested the deviation of this S/G ratio from a 1:1 ratio using a z- test. Second, to test spatial scale dependencies, we calculated S/G ratios at five *a priori*-defined spatial scales (0.1, 4, 16, 36, and 64 ha). In this study we established 30 plots at 200 m intervals. *A priori*-defined spatial scales corresponded to the area of each plot extended horizontally.

### Distribution exclusiveness among congener species

To evaluate the exclusivity of congeners, we calculated the checkerboard scores (C-scores; Stone & Roberts, 1990) for the genera with multiple species of the same genus distributed within the study area and which occurred in more than three plots (*Quercus*; wind pollinated, *Carpinus*; wind pollinated, and *Prunus*; insect pollinated). Note that there are differing opinions among researchers on whether the genus *Prunus* should be considered as a single genus or not (Ohba, 1992; Mabberley, 2008). Therefore, the genus *Prunus* in this study includes the subgenera *Cersus* and *Laurocerasus*. In this analysis, we analyzed six *Quercus* species, two *Carpinus* species, and two *Prunus* species.

We set *r*_*i*_ and *r*_*j*_ as the number of plots in which species *i* and *j*, respectively, were present; the checker unit *C*_*ij*_ associated with the two species was defined as follows:

(1) \begin{eqnarray*}{C}_{ij}= \left( {r}_{i}-{S}_{ij} \right) \times \left( {r}_{j}-{S}_{ij} \right) \end{eqnarray*}

where *S*_*ij*_ indicates the extent of co-occurrence (*i.e.,* the number of plots shared by the two species).

For *N* species, there are *P* = *N*(*N* − 1)/2 species pairs; thus, the C-score is calculated as follows:

(2) \begin{eqnarray*}C=\sum _{j=1}^{N}\sum _{i\lt J}{C}_{ij}/P\end{eqnarray*}

The C-score becomes larger as the two species occur more commonly across different plots. We simulated null models to compare the observed C-score with stochastic distributions. The null models, which were run 999 times for each species pair, randomly shuffles the number of species (α-diversity) among sampling locations while preserving the species occurrence totals (the plant density). All statistical analyses were performed using R software version 3.6.1 (R Core Team, 2019). EcoSimR package (Gotelli, Hart & Ellison, 2015) was used to compute C-score.

## Results

### *S/G* ratio

**Figure 2:**
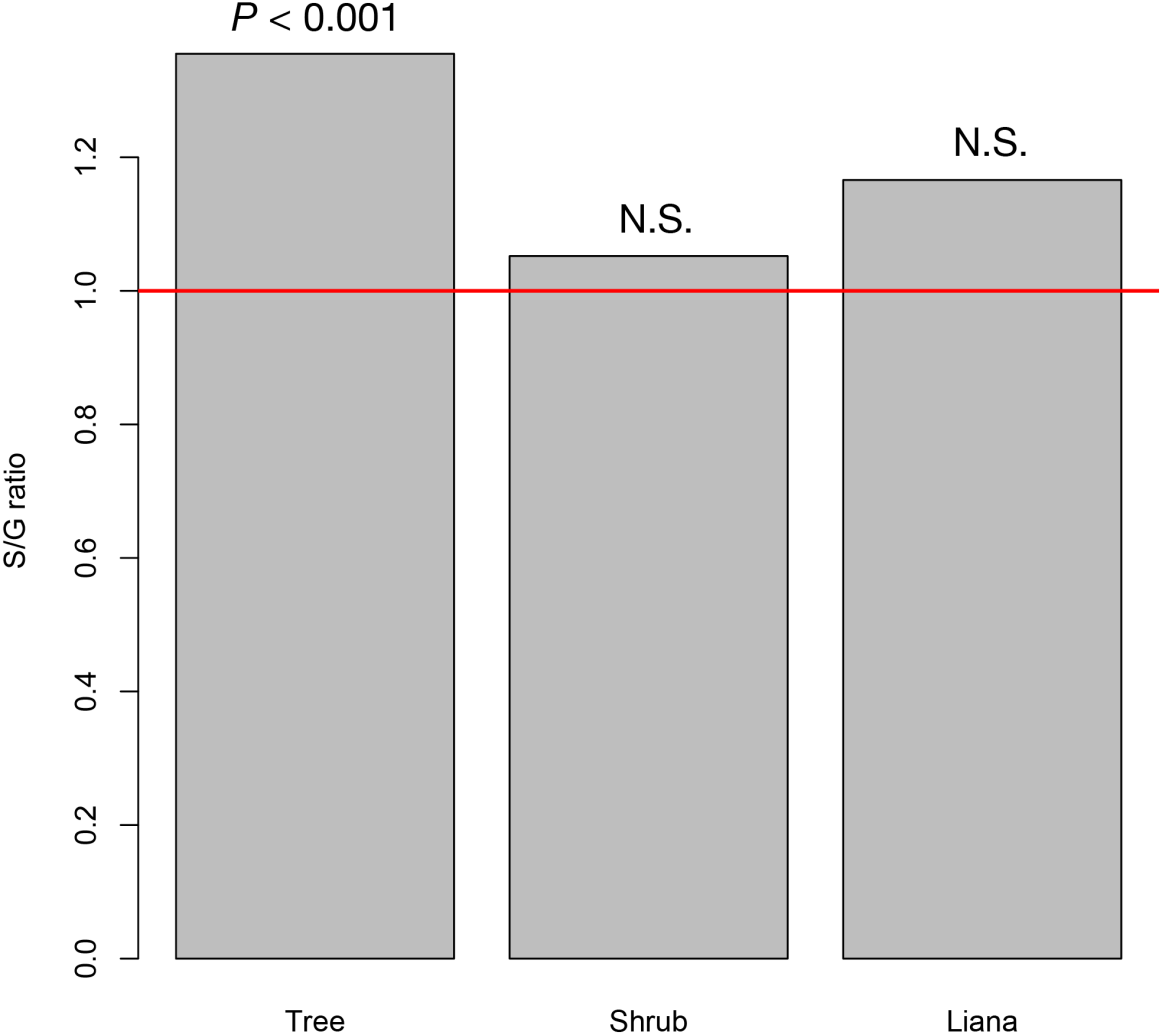
The species–to-genus ratios for the tree, shrub, and liana species categorized in the study site. This ratio significantly deviated from the 1:1 ratio (red horizontal line) for tree species but not shrub or liana species.

At the study site, we recorded 42 tree species (26 animal pollinated species and 16 wind pollinated species) from 31 genera, 20 shrub species (19 animal pollinated species and 1 wind pollinated species) from 19 genera, and seven liana species from six genera (seven animal pollinated species). The resultant S/G ratios for trees, shrubs, and lianas were 1.350, 1.021, and 1.200, respectively. Only the S/G ratio for tree species significantly deviated from the 1:1 ratio, whereas those for shrub and liana species did not (Fig. 2).

**Figure 3:**
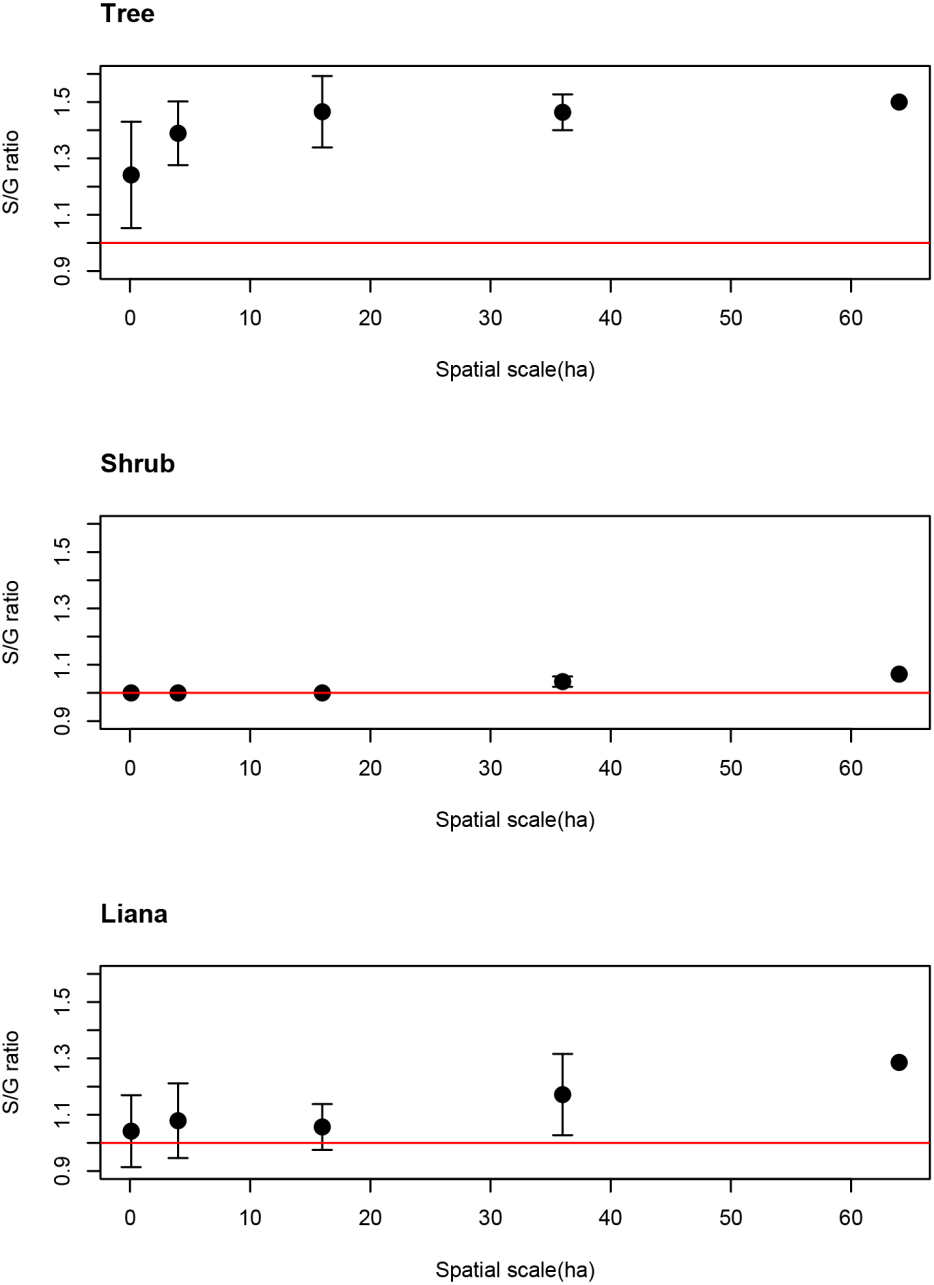
Spatial scale dependencies of species-to-genus ratios for the tree, shrub, and liana species categorized in the study site. Error bars represent standard deviations. Red lines indicate the 1:1 ratio.

The average S/G for trees increased as spatial scale increased. Even at the smallest spatial scale, however, the average S/G ratio for trees exceeded the 1:1 ratio (Fig. 3), indicating that the coexistence of congeners frequently occurs at the smallest spatial scale for tree species.

### C- score

**Figure 4:**
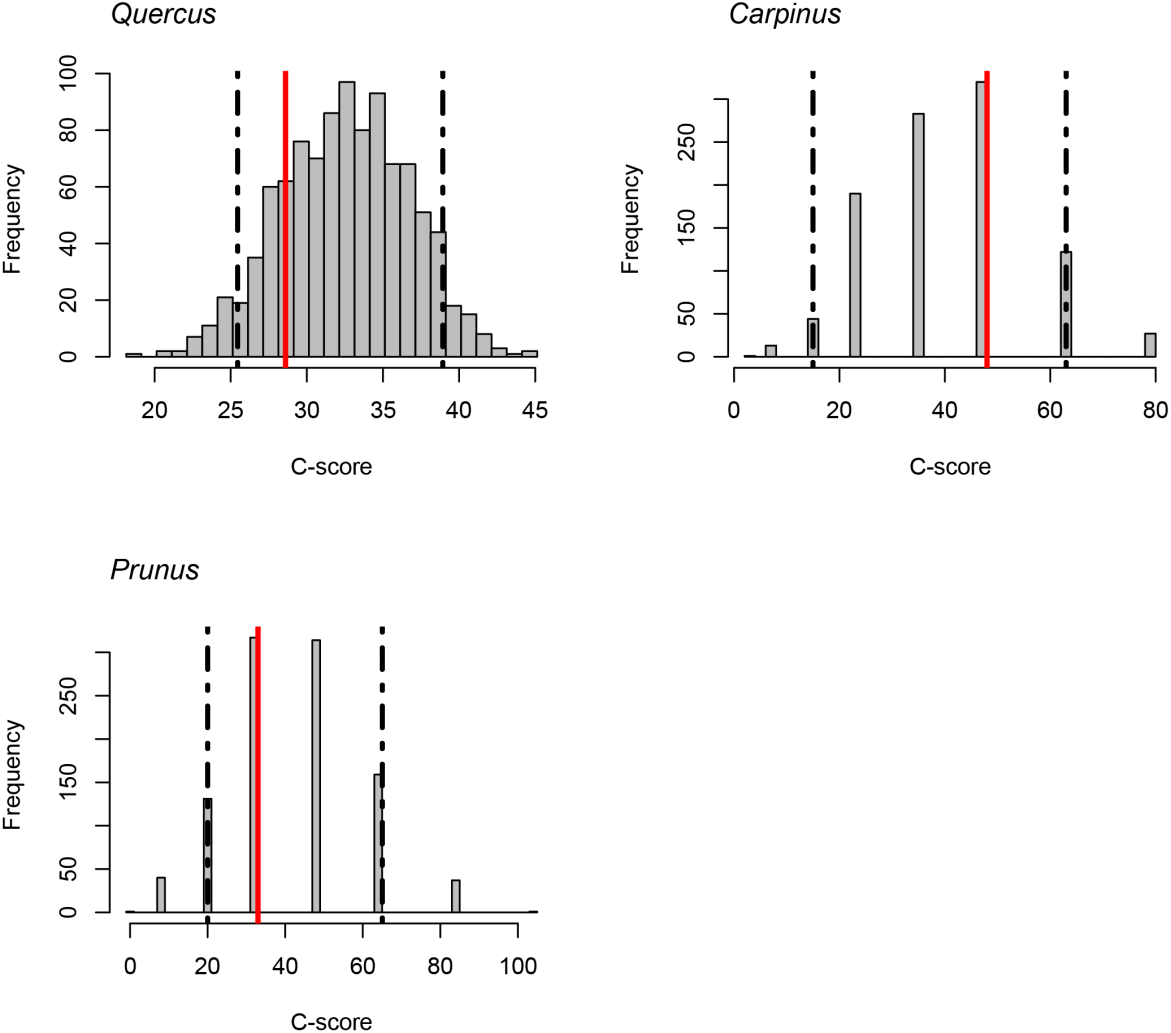
C-scores (checkerboard score; Stone & Roberts, 1990) for the genera *Quercus*, *Carpinus* and *Prunus*. Red lines indicate the observed C-score. Histograms indicate the null model distribution. Broken lines indicate the 95% (upper and lower) confidence intervals of the null model distribution.

The C-score did not fall outside the 95% confidence intervals of the null model distribution for *Quercus*, *Carpinus*, and *Prunus*. It indicates that statistically significant exclusive distribution of species from the same genus did not occur (Fig. 4).

## Discussion

Contrary to out prediction, our results show that, at least in our study area, closely related tree species often coexist even at the finest spatial scale and that statistically significant exclusive distribution of species from the same was not observed. These results suggest that co-occurrence of congeneric tree species is not prevented by reproductive interference. Previous theoretical studies predicted that reproductive interference could more readily prevent coexistence when compared to resource competition (Kuno, 1992; Kishi & Nakazawa, 2013). Additionally, empirical studies have demonstrated that reproductive interference can cause rapid congeneric species exclusion (Takakura et al., 2009; Takakura & Fujii, 2010; Runquist & Stanton, 2012). Therefore, our results did not meet the general expectation of reproductive interference effects on species coexistence, and thereby suggests that competitive exclusion among closely related tree species is somehow mitigated.

In contrast, the S/G ratio for shrubs and lianas did not deviate significantly from the 1:1 ratio, indicating that few congeneric shrub or liana species are distributed in the study area (∼1 km^2^). Since a low S/G ratio is generally a product of strong intrageneric competition (Elton, 1946), our results might imply the existence of competitive exclusion in shrub and liana species. However, it should be noted that the S/G ratio depends on the number of species present, and it would be expected to decrease in small communities regardless of competition levels (Gotelli & Colwell, 2001). In the study area, the deer population has recently increased, and this has affected the regeneration process and species richness of plant species (Shimoda et al., 1994; Maesako, 2015). As shrubs are likely to be more susceptible to deer grazing, a reduction in the number of shrub species due to deer grazing may have reduced the shrub S/G ratio. Nevertheless, it should be noted that closely related species of *Neolitsea aciculate*, a dominant shrub in our study area, are distributed about 15 km from the study site (Murata, 1977). This suggests that the low S/G ratio for shrubs and lianas would likely increase if the study area were to be extended by tens of kilometers.

In plants, the spatial extent of reproductive interference corresponds to pollen transfer distance (Takakura et al., 2011); consequently, coexistence of closely related plant species is expected at the spatial extent to which pollen flow does not occur. However, the frequent pollen dispersal range of the dominant tree genera in our study site (*i.e., Quercus*, *Acer*, and *Machilus*) is within a few tens of meters (Nakanishi et al., 2004; Kikuchi et al., 2009; Watanabe et al., 2018). This suggests that the congeneric tree species in our study site coexist on spatial scales at which reproductive interference can occur frequently and that, given the spatial factor, the effects of reproductive interference could not be avoided in this study. A previous theoretical study suggested that recruitment fluctuation could enable coexistence of closely related tree species on a local scale by producing temporal resource partitioning (a mechanism known as the storage effect) (Usinowicz, Wright & Ives, 2012). One of the dominant genera in our study area, *Quercus*, shows considerable variation in annual seed production (Hirayama et al., 2012), which might contribute to maintaining the coexistence of congener species. Another mechanism that could potentially weaken reproductive interference is reproductive character displacement (Pfennig & Pfennig, 2009). Eaton et al. (2012) showed that disparity in the floral traits of plants could reduce negative reproductive interactions among closely related species. However, there remains a lack of direct evidence to show that reproductive character displacement reduces the effect of reproductive interference. Moreover, experimental evidence of reproductive interference is limited to herbaceous plants.

Previous studies reveal that specialist natural enemies, such as herbivores and pathogens, maintain tree species diversity by the reducing survival rates of conspecific seeds and seedlings located close to reproductive adults or in high conspecific density areas (Janzen–Connell effect; Janzen, 1970; Seiwa et al., 2008; Comita et al., 2014). Additionally, coexistence theory (Chesson, 2000; Barabás, Stump & Stump, 2018) suggests that negative frequency-dependence is essential for stable coexistence of species (Levine & HilleRisLambers, 2009). In contrast, reproductive interference involves positive frequency-dependent selection, it can rapidly lead to species exclusion ( Kishi & Nakazawa, 2013) and eventually hamper species coexistence. Therefore, mitigation of reproductive interference is important to coexist with closely related species (Katsuhara & Ushimaru, 2019). Since positive and negative frequency-dependent processes possibly act simultaneously in nature, it is necessary to compare the relative importance of the two processes in the future.

In future research, investigation on a larger spatial scale with a more complex analysis will be required to determine the relationship between plant life history and the spatial scale of exclusive distribution. Previous studies on herbaceous plants suggest that reproductive interference plays an important role in community assembly (Eaton et al., 2012; Whitton, Sears & Maddison, 2017); however, our results, in concert with prior studies, indicate that reproductive interference is somewhat less effective than expected, especially in long-lived plant species. In this study, we mainly focused on competitive interaction but it has been proposed that shared floral displays might increase reproductive success (facilitation) and that co-flowering plants may, instead of competing, facilitate pollination (Ghazoul, 2006). A study in an alpine plant community reports that facilitative and neutral pollinator-mediated interactions among plants prevailed over competition (Tur et al., 2016). Additionally, recent studies have reported that the interplay between competition and facilitation affects local population persistence (Losapio et al., 2021). Such facilitative interactions might affect the coexistence of closely related species. Further study is therefore necessary to identify the key life history traits that mitigate the effects of reproductive interference.

## Supplemental Information

10.7717/peerj.12150/supp-1 Supplemental Information 1 Species list for this study

10.7717/peerj.12150/supp-2 Supplemental Information 2 Raw data and code for Fig. 3

10.7717/peerj.12150/supp-3 Supplemental Information 3 Raw data and code for Fig. 4

10.7717/peerj.12150/supp-4 Supplemental Information 4 Raw data

## Additional Information and Declarations

### Field Study Permissions

The following information was supplied relating to field study approvals (i.e., approving body and any reference numbers): Field permission provided by Nagatani Village Hiroshi, Nagatani Park Office (6-6).

### Data Availability

The following information was supplied regarding data availability: The raw measurements and code are available in the Supplemental Files.
