# Supplementary material for: Co-occurrence pattern of congeneric tree species provides conflicting evidence for competition relatedness hypothesis
Source: PeerJ. 2021 Nov 2;9:e12150. doi: 10.7717/peerj.12150 (PMC8570171; doi:10.7717/peerj.12150)
Supplement: Supplemental Information 1 [file peerj-09-12150-s001.docx]

Species list

Pinaceae

Abies firma Siebold et Zucc.

Tsuga sieboldii Carrière

Podocarpaceae

Nageia nagi (Thunb.) Kuntze

Cupressaceae

Chamaecyparis obtusa (Siebold et Zucc.) Endl.

Cryptomeria japonica (L.f.) D.Don

Taxaceae

Torreya nucifera (L.) Siebold et Zucc.

Schisandraceae

Illicium anisatum L.

Lauraceae

Cinnamomum yabunikkei H.Ohba

Lindera erythrocarpa Makino

Litsea coreana H.Lév.

Machilus japonica Siebold et Zucc. ex Blume

Neolitsea aciculata (Blume) Koidz.

Lardizabalaceae

Akebia trifoliata (Thunb.) Koidz.

Stauntonia hexaphylla (Thunb.) Decne.

Vitaceae

Parthenocissus tricuspidata (Siebold et Zucc.) Planch.

Vitis ficifolia Bunge

Vitis flexuosa Thunb.

Nekemias cantoniensis (Hook. et Arn.) J.Wen et Z.L.Nie

Fabaceae

Biancaea decapetala (Roth) O.Deg.

Wisteria floribunda (Willd.) DC.

Rosaceae

Aria japonica Decne.

Prunus (Cerasus) jamasakura (Siebold ex Koidz.) H.Ohba

Prunus (Laurocerasus) spinulosa (Siebold et Zucc.) C.K.Schneid.

Photinia glabra (Thunb.) Maxim.

Rosa multiflora Thunb.

Elaeagnaceae

Elaeagnus pungens Thunb.

Rhamnaceae

Berchemia racemosa Siebold et Zucc.

Ulmaceae

Zelkova serrata (Thunb.) Makino

Cannabaceae

Aphananthe aspera (Thunb.) Planch.

Celtis sinensis Pers.

Moraceae

Morus australis Poir.

Fagaceae

Castanopsis cuspidata (Thunb.) Schottky

Quercus acuta Thunb.

Quercus gilva Blume

Quercus glauca Thunb.

Quercus myrsinifolia Blume

Quercus salicina Blume

Quercus sessilifolia Blume

Myricaceae

Morella rubra Lour.

Betulaceae

Carpinus laxiflora (Siebold et Zucc.) Blume

Carpinus tschonoskii Maxim.

Ostrya japonica Sarg.

Euphorbiaceae

Mallotus japonicus (L.f.) Müll.Arg.

Triadica sebifera (L.) Small

Anacardiaceae

Rhus javanica L. var. chinensis (Mill.) T.Yamaz.

Sapindaceae

Acer palmatum Thunb.

Acer rufinerve Siebold et Zucc.

Sapindus mukorossi Gaertn.

Simaroubaceae

Ailanthus altissima (Mill.) Swingle

Meliaceae

Melia azedarach L.

Rutaceae

Zanthoxylum ailanthoides Siebold et Zucc.

Zanthoxylum piperitum (L.) DC.

Cornaceae

Cornus macrophylla Wall.

Ternstroemiaceae

Cleyera japonica Thunb.

Eurya japonica Thunb. var. japonica

Theaceae

Camellia japonica L.

Symplocaceae

Symplocos prunifolia Siebold et Zucc.

Clethraceae

Clethra barbinervis Siebold et Zucc.

Ericaceae

Pieris japonica (Thunb.) D.Don ex G.Don subsp. japonica

Vaccinium bracteatum Thunb.

Apocynaceae

Trachelospermum asiaticum (Siebold et Zucc.) Nakai

Oleaceae

Osmanthus heterophyllus (G.Don) P.S.Green

Lamiaceae

Callicarpa japonica Thunb.

Callicarpa mollis Siebold et Zucc.

Clerodendrum trichotomum Thunb.

Aquifoliaceae

Ilex integra Thunb.

Ilex micrococca Maxim.

Ilex pedunculosa Miq.

Ilex rotunda Thunb.
